# Estimated Resource Costs for Implementation of CDC’s Recommended COVID-19 Mitigation Strategies in Pre-Kindergarten through Grade 12 Public Schools — United States, 2020–21 School Year

**DOI:** 10.15585/mmwr.mm6950e1

**Published:** 2020-12-18

**Authors:** Ketra L. Rice, Gabrielle F. Miller, Fátima Coronado, Martin I. Meltzer

**Affiliations:** 1 CDC COVID-19 Response Team.

*On December 11, 2020, this report was posted online as an *MMWR *Early Release.*

As school districts across the United States consider how to safely operate during the 2020–21 academic year, CDC recommends mitigation strategies that schools can adopt to reduce the risk for transmission of SARS-CoV-2, the virus that causes coronavirus disease 2019 (COVID-19) (*1*). To identify the resources and costs needed to implement school-based mitigation strategies and provide schools and jurisdictions with information to aid resource allocation, a microcosting methodology was employed to estimate costs in three categories: materials and consumables, additional custodial staff members, and potential additional transportation. National average estimates, using the national pre-kindergarten through grade 12 (preK–12) public enrollment of 50,685,567 students, range between a mean of $55 (materials and consumables only) to $442 (all three categories) per student. State-by-state estimates of additional funds needed as a percentage of fiscal year 2018 student expenditures (*2*) range from an additional 0.3% (materials and consumables only) to 7.1% (all three categories); however, only seven states had a maximum estimate above 4.2%. These estimates, although not exhaustive, highlight the level of resources needed to ensure that schools reopen and remain open in the safest possible manner and offer administrators at schools and school districts and other decision-makers the cost information necessary to budget and prioritize school resources during the COVID-19 pandemic.

Approximately 50 million students are enrolled in public elementary and secondary schools in the United States (*3*); since March 2020, approximately 270,000 cases of COVID-19 have been reported among school-aged children (aged 5–17 years) (*4*). Although current evidence indicates that the risk for SARS-CoV-2–related hospitalizations and deaths among children is lower than that among adults, the risk for morbidity and mortality posed to teachers and other staff members in the school environment is expected to mirror that of other adults with similar demographic characteristics in the community (*5*). As school districts across the United States consider how to safely operate schools for the 2020–21 academic year, CDC provides indicators to help local jurisdictions determine the risk associated with operating schools for in-person learning. These indicators include measures of underlying community transmission and a measure of adherence to five primary mitigation strategies (*1*): 1) consistent and correct use of masks, 2) social distancing to the extent possible, 3) hand hygiene and respiratory etiquette, 4) cleaning and disinfection, and 5) contact tracing in collaboration with the local health department. Other mitigation strategies that can also be used concurrently include cohorting and staggered scheduling (*1*). In this analysis, the resources needed to implement four of the five key mitigation strategies were identified and costs estimated, with the goal of providing estimates to aid resource allocation to ensure the safe operation of schools and reduce school-based transmission of SARS-CoV-2. Contact tracing, although an essential strategy to reduce transmission, was excluded because those costs are not financed by school district budgets.

A microcosting approach (*6*) was used to estimate resources and costs associated with implementing the critical CDC-recommended mitigation strategies. This approach involves collecting detailed data on resources needed for each strategy and applying unit costs for those resources. From a school budget perspective, resources needed to implement the four strategies are identified (Supplementary Table, https://stacks.cdc.gov/view/cdc/97907), and total costs associated with each resource are estimated. Direct budgetary costs are the focus of this analysis; opportunity costs are excluded. The estimates indicate resources needed in addition to those already allocated in annual school budgets. Costs were aggregated and analyzed nationally and for each state and the District of Columbia (DC). A range is provided for each cost to indicate levels of cost variation around each estimate.

Personnel costs for school custodians were estimated using data from the Bureau of Labor Statistics.* To account for fringe benefits, annual wages were increased by the state and local government average of 37.8%.^†^ Labor demand for school custodians was derived from a study of national standards for allocating school custodians that increases the recommended number of custodians from a tier 3 level of cleaning (one custodian per 28,000–31,000 ft^2^) to a tier 2 level of cleaning (one custodian per 18,000–20,000 ft^2^) (*7*). The American Federation of Teachers estimates that tier 2 cleaning is needed for an estimated 10% of targeted physical areas per school (i.e., bathrooms, food service areas, and high-need classrooms, including special needs classes) (*8*). To allow for variation in school size, ranges of estimates for additional custodial services were estimated. The low estimate used an additional 1.25 full time equivalents (FTEs), and the high estimate used 2.5 FTEs (Supplementary Table, https://stacks.cdc.gov/view/cdc/97907) (*7*). Costs are inflated and reported in 2020 U.S. dollars.^§^ Potential additional transportation costs were extrapolated from a report by the American Federation of Teachers that forecasts an estimated 36% national increase in funding needed for school transportation (*7*). These potential costs assume that some schools would require additional buses, drivers, and protocols to implement social distancing on buses. The 36% national increase was distributed across states and adjusted by states’ past year transportation spending per student (*8*). Ranges for nonlabor costs for all materials and consumables were obtained from the U.S. General Services Administration (GSA) Supply Catalog 2020, GSA Advantage Disaster Relief and Pandemic Products online catalog, and through various e-commerce marketplace websites to derive a range of cost estimates across multiple sources and reflect price variability for materials across vendors.^¶^ Aggregated material costs were adjusted for each state using the 2020 state-based composite cost of living index.** Average costs per student were calculated using the national preK–12 public student enrollment of 50,685,567 students (*3*). State-based cost estimates were adjusted based on the number of schools and the total school population within each state. Estimated pandemic-related per-student costs were calculated as a percentage of fiscal year 2018 per-student expenditures as reported by the National Center for Education Statistics (*2*).

National costs per student range between a mean of $55 (materials and consumables only) to $442 (three categories) (Table 1). The highest cost categories were related to employing additional custodians per school (44.8% of total costs) and potential additional transportation (42.8% of total cost). For state-based estimates, the incremental increase in costs per student for materials and consumables ranges from $47 to $109 per student; implementation of all strategies combined (including high and low projections for additional custodial staff members) range between $204 (Utah) and $912 (DC) (Table 2). Utah’s and DC’s average total costs are lower and higher, respectively, than the national range (Table 1) because of their lower and higher transportation costs per pupil, relative to other states. All other state estimates fall within the national range. Additional funds needed as a percentage of fiscal year 2018 per-student expenditures range from 0.3% (materials and consumables only) to 7.1% (all three categories), although only seven states had a maximum estimate >4.2% (Table 2).

**TABLE 1:** Estimated national costs for selected resources needed for school-based implementation of CDC's recommended COVID-19 mitigation strategies — United States, 2020–21 school year

Cost item	Unit cost, USD*	No. of units^†^	Total cost, USD	Unit cost range, USD*	Total cost range, USD
**Materials and consumables**					
Plexiglass shield (1 per school)	74.99	98,456	7,383,215	49.50–147.75	4,873,572–19,272,762
Student desk shields (1 per student)	37.20	50,685,567	1,885,503,092	14.99–75.95	759,776,649–3,849,568,814
Reusable face shield (1 per teacher and other staff member)	4.88	6,382,813	31,148,128	1.93–17.40	12,318,829–111,060,947
Disposable face masks (1-month supply per student, teacher, and staff member)	0.31	1,141,367,601	353,823,956	0.10–1.50	114,136,760–1,712,051,402
Disposable gloves (2 pair per teacher and other staff member)	0.18	12,765,626	2,297,813	0.17–0.25	2,170,156–3,191,407
Hand sanitizer dispenser (4 units per school)	109.89	393,824	43,277,319	81.67–137.36	32,163,606–54,095,665
Hand sanitizer dispenser refills (1 refill per month per unit per school)	2.07	3,938,240	8,163,972	1.55–2.58	6,104,272–10,160,659
Hand sanitizer (1 bottle per student)	4.89	50,685,567	247,852,423	3.67–6.11	186,016,031–309,688,814
Multipurpose cleaners (180-day supply per school)	4.48	17,722,080	79,394,918	3.36–5.60	59,546,189–99,243,648
Disinfectants/Virucides (180-day supply per school)	4.99	17,722,080	88,344,569	3.74–6.24	66,280,579–110,585,779
No touch thermometer (2 per school)	59.00	196,912	11,617,808	25.99–75.99	5,117,743–14,963,343
Oximeter (2 per school)	84.99	196,912	16,735,551	15.95–199.99	3,140,746–39,380,431
Signage (1 kit of 100 hallway floor signs and 30 hallway directional arrows per school)	268.44	98,456	26,429,529	178.96–357.92	18,604,246–35,239,372
**Total materials and consumables^§^**	—	—	**2,801,972,293**	—	**1,075,901,224–12,584,162,010**
**Personnel** ^¶^					
Custodian FTEs (high estimate)**	40,837	246,140	10,051,712,049	31,314–51,953	7,707,613,702–12,699,213,527
Custodian FTEs (low estimate) **	40,837	123,070	5,025,824,797	31,314–51,953	3,853,806,851–6,349,606,764
**Potential additional transportation^††^**	—	—	9,600,000,000	—	8,131,200,000–18,969,600,000
**Cost per student** ^§§^					
Average materials and consumables cost per student	—	—	55	—	21–248
Average personnel cost per student (high)	—	—	198	—	152–251
Average personnel cost per student (low)	—	—	99	—	76–125
Average potential transportation cost per student^§§^	—	—	189	—	160–374

**Abbreviations:** COVID-19 = coronavirus disease 2019; FTEs = full-time equivalents; USD = U.S. dollars.

* Unit cost is the average cost per resource and the unit cost range are minimum and maximum cost values per resource derived from all material sources, retrieved from U.S. General Services Administration (GSA) Global Supply Catalog (https://www.gsaglobalsupply.gsa.gov/advantage/), GSA Advantage Disaster (https://www.gsaadvantage.gov/advantage/) Disaster Relief and Pandemic Products Supply; School Kids Healthcare (https://www.buyemp.com/school-kids-healthcare-transition), School Health (https://www.schoolhealth.com), School Nurse Supply (https://www.schoolnursesupplyinc.com), and Amazon Marketplace (https://www.amazon.com/s?k=coronavirus).

^†^ Quantity of units for schools and school populations derived from school population fiscal year 2018 data published by the National Center for Education Statistics. https://nces.ed.gov/.

^§^ Cost range for materials and consumables adjusted by lowest and highest state composite cost of living index. https://meric.mo.gov/data/cost-living-data-series.

^¶^ Cost for personnel derived from the Bureau of Labor Statistics wage estimates (updated as of May 2019) and are inflation-adjusted to August 2020 USD using the Consumer Price Index (CPI) Databases (https://www.bls.gov/cpi/data.htm); all other costs reported in current 2020 USD.

** The high and low estimates for school custodians are 2.5 and 1.25 additional custodian FTEs per school for tier 2 cleaning needed for an estimated 10% of targeted physical areas per school (i.e., bathrooms, food service areas, and high need classrooms, including special needs classes) https://nces.ed.gov/pubs2003/2003347.pdf; https://www.aft.org/sites/default/files/wysiwyg/reopen-schools-financial-implications.pdf.

^††^ Costs of potential additional transportation, where needed, were estimated assuming that such costs are equivalent to 36% of national costs for student transportation. https://www.aft.org/sites/default/files/wysiwyg/reopen-schools-financial-implications.pdf.

^§§^ Based on national pre-kindergarten–grade 12 public student enrollment of 50,685,567 students. https://nces.ed.gov/.

**TABLE 2:** Estimated costs for selected resources needed for school-based implementation of CDC's recommended COVID-19 mitigation strategies, by state — United States, 2020–21 school year

State	No. of schools*	No. of teachers/staff members*	Total student enrollment*	Cost, USD							Pandemic costs, % increase,** range
				Materials/ Consumables	Custodian FTEs (low est.)^†^	Custodian FTEs (high est.)^†^	Potential additional transportation^§^	Avg. total cost per student, range^¶^	Avg. cost per student (materials/consumables only)	FY18* expenditures per student	
Alabama	1,509	71,628	742,444	36,773,905	63,369,776	126,739,552	136,505,880	319–404	50	9,717	0.5–4.2
Alaska	508	16,982	132,872	9,431,191	30,381,042	60,762,083	29,442,960	521–750	71	17,726	0.4–4.2
Arizona	2,284	103,175	1,110,851	61,469,251	111,849,022	223,698,043	136,946,520	279–380	55	8,373	0.7–4.5
Arkansas	1,088	73,658	496,085	24,187,597	46,814,518	93,629,037	68,657,040	282–376	49	10,168	0.5–3.7
California	10,303	577,836	6,304,266	497,309,250	618,835,013	1,237,670,026	638,704,080	278–377	79	12,664	0.6–3.0
Colorado	1,862	111,939	910,280	51,930,527	95,898,121	191,796,241	99,713,880	272–377	57	10,238	0.6–3.7
Connecticut	1,369	98,166	531,288	36,477,044	84,231,421	168,462,843	189,668,880	584–743	69	20,147	0.3–3.7
Delaware	223	17,098	136,293	8,114,416	11,223,913	22,447,827	37,752,480	419–501	60	15,282	0.4–3.3
District of Columbia	228	14,106	87,315	7,756,625	13,773,041	27,546,082	44,319,600	754–912	89	23,155	0.4–3.9
Florida	4,322	345,644	2,832,424	155,323,788	197,729,771	395,459,542	381,070,800	259–329	55	9,663	0.6–3.4
Georgia	2,297	224,488	1,768,642	87,993,477	97,806,719	195,613,439	323,339,040	288–343	50	10,760	0.5–3.2
Hawaii	290	22,596	180,837	19,753,419	16,819,007	33,638,014	23,746,680	334–427	109	15,242	0.7–2.8
Idaho	744	27,186	301,186	15,700,537	33,499,456	66,998,911	38,018,160	290–401	52	7,846	0.7–5.1
Illinois	4,175	260,463	2,005,153	99,427,837	221,424,361	442,848,721	506,354,760	413–523	50	15,912	0.3–3.3
Indiana	1,921	152,826	1,054,187	52,156,534	$92,649,830	185,299,660	234,095,040	359–447	49	10,033	0.5–4.5
Iowa	1,349	72,886	511,850	25,861,692	67,664,761	135,329,522	77,619,960	334–467	51	11,724	0.4–4.0
Kansas	1,320	73,271	497,088	23,769,356	61,662,744	123,325,488	81,582,480	336–460	48	11,095	0.4–4.1
Kentucky	1,541	97,712	680,978	35,461,085	71,535,339	143,070,678	148,091,760	375–480	52	11,081	0.5–4.3
Louisiana	1,390	107,600	715,135	36,963,040	54,206,386	108,412,772	170,258,760	366–441	52	11,636	0.4–3.8
Maine	611	35,241	180,473	11,492,983	33,131,047	66,262,095	48,734,640	517–701	64	15,069	0.4–4.7
Maryland	1,437	115,516	893,684	63,235,650	72,450,055	144,900,111	255,236,040	437–518	71	15,155	0.5–3.4
Massachusetts	1,862	128,291	964,791	69,173,739	118,317,113	236,634,225	282,796,200	487–610	72	18,328	0.4–3.3
Michigan	3,468	181,468	1,516,398	75,527,660	169,352,411	338,704,821	270,858,960	340–452	50	11,688	0.4–3.9
Minnesota	2,478	117,236	884,944	49,506,741	140,130,095	280,260,189	237,747,960	483–641	56	12,910	0.4–5.0
Mississippi	1,076	67,757	478,321	22,396,022	41,497,850	82,995,700	75,434,040	291–378	47	8,909	0.5–4.2
Missouri	2,424	128,938	915,472	44,686,239	117,494,068	234,988,135	191,587,680	386–515	49	11,034	0.4–4.7
Montana	823	21,329	149,474	8,940,482	41,734,659	83,469,318	29,641,320	537–817	60	11,512	0.5–7.1
Nebraska	1,085	47,292	323,766	16,609,144	53,805,991	107,611,982	43,724,880	353–519	51	12,813	0.4–4.0
Nevada	662	26,430	485,785	29,217,364	36,329,799	72,659,597	61,859,880	262–337	60	9,040	0.7–3.7
New Hampshire	490	31,981	179,433	10,712,581	25,540,197	51,080,393	48,024,360	470 –612	60	16,588	0.4–3.7
New Jersey	2,588	236,559	1,408,102	95,276,011	145,057,788	290,115,576	434,831,040	479–583	68	20,316	0.3–2.9
New Mexico	884	37,573	334,345	16,116,820	38,021,569	76,043,139	38,696,040	278–391	48	9,963	0.5–3.9
New York	4,824	372,692	2,724,663	234,815,599	302,459,976	604,919,952	1,042,712,640	580–691	86	23,686	0.4–2.9
North Carolina	2,603	190,855	1,553,513	82,013,680	111,732,994	223,465,988	218,620,080	265–337	53	9,277	0.6–3.6
North Dakota	518	17,984	111,920	5,970,395	28,284,484	56,568,967	22,965,480	511–764	53	13,783	0.4–5.5
Ohio	3,619	322,611	1,704,399	86,775,862	179,032,654	358,065,308	381,186,000	380–485	51	12,893	0.4–3.8
Oklahoma	1,800	85,914	695,092	33,314,203	76,148,280	152,296,560	64,328,040	250–360	48	8,174	0.6–4.4
Oregon	1,242	65,928	608,014	45,139,592	67,047,072	134,094,145	112,797,000	370–480	74	11,903	0.6–4.0
Pennsylvania	3,019	241,548	1,726,809	98,512,658	154,654,766	309,309,532	486,006,840	428–518	57	16,377	0.3–3.2
Rhode Island	313	19,482	142,949	9,372,034	16,918,292	33,836,583	38,119,680	451–569	66	16,954	0.4–3.4
South Carolina	1,248	78,108	777,507	41,046,461	51,721,301	103,442,602	116,117,640	269–335	53	10,705	0.5–3.1
South Dakota	698	19,543	137,823	7,595,999	32,185,705	64,371,410	18,681,120	424–658	55	10,263	0.5–6.4
Tennessee	1,859	128,469	1,001,967	49,849,862	79,092,549	158,185,099	130,509,000	259–338	50	9,599	0.5–3.5
Texas	8,826	690,078	5,401,341	273,803,482	391,623,742	783,247,483	564,675,120	228–300	51	9,670	0.5–3.1
Utah	1,033	56,146	668,274	35,796,979	45,622,342	91,244,683	54,689,400	204–272	54	7,576	0.7–3.6
Vermont	314	18,183	88,028	5,678,841	17,751,189	35,502,379	21,240,720	507–709	65	20,149	0.3–3.5
Virginia	2,133	178,550	1,291,462	72,391,508	99,604,648	199,209,295	297,275,760	363–440	56	12,224	0.5–3.6
Washington	2,427	94,882	1,110,367	68,869,580	153,215,600	306,431,200	205,493,040	385–523	62	12,985	0.5–4.0
West Virginia	744	38,452	272,266	13,906,999	34,614,395	69,228,791	86,911,560	497–625	51	11,572	0.4–5.4
Wisconsin	2,255	101,250	860,753	45,536,382	112,992,469	225,984,938	162,415,440	373–504	53	12,446	0.4–4.1
Wyoming	370	17,268	94,258	4,924,000	19,483,025	38,966,051	26,206,560	537–744	52	16,134	0.3–4.6
**Total costs^††^**	**—**	**—**	**—**	**3,014,066,119**	**4,998,422,363**	**9,996,844,725**	**9,436,012,920**	**—**	**—**	**—**	**—**

**Abbreviations:** avg. = average; COVID-19 = coronavirus disease 2019; est. = estimate; FTEs = full-time equivalents; FY = fiscal year; USD = U.S. dollars.

* Number of schools, number of teachers and staff members, total student enrollment, and FY18 expenditures per student derived from school population FY18 data published by the National Center for Education Statistics. https://nces.ed.gov/.

^†^ The high and low estimates for school custodians are 2.5 and 1.25 additional custodian FTEs per school for tier 2 cleaning needed for an estimated 10% of targeted physical areas per school (i.e., bathrooms, food service areas, and high-need classrooms, including special needs classes). https://nces.ed.gov/pubs2003/2003347.pdf; https://www.aft.org/sites/default/files/wysiwyg/reopen-schools-financial-implications.pdf.

^§^ Costs of potential additional transportation, where needed, were estimated assuming that such costs are equivalent to a 36% increase of FY18 state expenditures for student transportation. https://www.aft.org/sites/default/files/wysiwyg/reopen-schools-financial-implications.pdf.

^¶^ Low percentage cost calculated using only the average cost per student for materials and consumables. High percentage cost calculated using high average total cost per student, which includes all three cost categories (Materials and Consumables, Custodian FTEs [high est.], and Potential additional transportation).

** Percentage increase in expenditure per student above FY18 levels.

^††^ Total costs for each category of state estimates fall within the national range of estimates per category. The national range uses a range of prices nationwide for that item, multiplied by the number of units nationally, adjusted by the highest and lowest nationwide cost of living index. The state estimates are specific to each state’s school population and are estimated using a combination of past year transportation expenditures, the average wage for custodians, and average price of materials in that state, with adjustments for the state-specific cost of living index.

## Discussion

Successfully operating schools during the COVID-19 pandemic requires sufficient resources to implement and sustain effective mitigation strategies. This cost estimate for the resources needed to safely reopen and keep schools open for in-person learning found that the average school district will need to invest $55 per student for materials and consumables only. This cost increases to a maximum average of $442 per student if a school district needs or chooses to employ the maximum number of additional custodial staff members per school and add additional transportation. Costs might be lower, depending on the extent of the learning model as schools transition from virtual to hybrid or in-person learning. These estimates provide schools, districts, and other jurisdictions with the cost information necessary to budget and prioritize resources during the COVID-19 pandemic.

The findings in this report are subject to at least four limitations. First, costs related to food service operations were not included. Although some schools might incur additional costs to provide student meals, estimates might significantly vary given differences in the need for school meal programs across districts. Second, a 1-month supply of face masks for the school population was estimated and was not included as an ongoing cost for schools, based on the assumption that teachers and staff members would purchase their own masks, and schools would add masks to the student supply list. Third, costs related to social distancing within the classroom were not estimated because other resources for schools recommended by CDC (e.g., physical barriers in the classroom, such as individual student desk shields) (*9*) were included in the estimates. Resource needs and costs for social distancing will vary with individual school needs. Finally, although contact tracing is a primary mitigation strategy, costs for contact tracing were excluded because school districts do not bear the financial responsibility for hiring and employing contact tracers.

The benefits of schools extend beyond academic achievements and have critical implications for student health, safety, social and emotional well-being, and the economy, because in-person learning allows parents and caretakers to return to work (*9*). Although the list of resources identified in this analysis is not exhaustive, the cost estimates illustrate the level of resources needed to help ensure that schools both reopen and operate in the safest possible manner. In addition, this report provides cost data that can be used as a baseline for future studies examining the cost-effectiveness of mitigation strategies in school settings and those comparing costs and benefits across multiple sectors of the economy.

**Summary**

**What is already known about this topic?**

CDC recommends mitigation strategies that schools can adopt to minimize the risk for transmission of SARS-CoV-2 in school settings.

**What is added by this report?**

Costs per student for implementation of strategies range from a mean of $55 (materials and consumables only) to $442 (materials and consumables, additional custodial staff members, and potential additional transportation). Incremental costs across states range from an additional 0.3% to 7.1% in costs needed above reported fiscal year 2018 school expenditures per student.

**What are the implications for public health practice?**

These findings offer schools, school districts, and other decision makers cost information necessary to budget and prioritize school resources during the COVID-19 pandemic.

## Footnotes

* The Bureau of Labor Statistics (BLS) provides national and state occupational employment and wage estimates each May (in the given year); the latest published estimates are provided for May 2019. https://www.bls.gov/oes/current/oessrcst.htm.

† Employer Costs for Employee Compensation reports are provided by BLS and provide benefit rates by industry type. https://www.bls.gov/bls/news-release/ecec.htm#2019.

§ Consumer Price Index databases are provided annually by BLS. https://www.bls.gov/cpi/data.htm.

¶ Nonlabor material sources retrieved from U.S. General Services Administration (GSA) Global Supply Catalog 2020 (https://www.gsaglobalsupply.gsa.gov/advantage/); GSA Advantage (https://www.gsaadvantage.gov/advantage) Disaster Relief and Pandemic Products Supply; School Kids Healthcare (https://www.buyemp.com/school-kids-healthcare-transition), School Health (https://www.schoolhealth.com), School Nurse Supply (https://www.schoolnursesupplyinc.com), and Amazon Marketplace (https://www.amazon.com/s?k=coronavirus).

** The Missouri Economic Research and Information Center Cost of Living Data Series provides state level cost of living indices for 2020. https://meric.mo.gov/data/cost-living-data-series.
